# Advancing Inclusive Design Practice for Queer Youth Using Digital Technologies for Mental Health

**DOI:** 10.5694/mja2.70183

**Published:** 2026-04-14

**Authors:** Adam Poulsen, Ian B. Hickie, Samuel J. Hockey, Frank Iorfino, Haley M. LaMonica

**Affiliations:** ^1^ Brain and Mind Centre University of Sydney Sydney New South Wales Australia

**Keywords:** eHealth, gender identity, healthcare disparities, mental health services, research design, social determinants of health, social justice, technology

## Abstract

Youth mental ill health has increased worldwide, and non‐inclusive technology design practice risks overlooking queer youth. This article calls for greater inclusivity and equity in the design of digital technologies used by queer youth to access mental health resources, support and service pathways across diverse global contexts, including internet‐based tools, digital platforms and digitally enabled therapeutic interventions, such as mobile applications (apps), online services, chatbots and social media. It warns of threats such as persistent invisibility and prejudiced technology design practice markedly affecting mental health equity and access for queer youth. It proposes three key principles to guide practitioners and researchers in designing digital technologies used by queer youth for mental health: (i) partner and design with queer young people; (ii) embed queer theory into research and design; and (iii) design for sustainability. The principles and recommendations presented here are also relevant to other digital health fields and practitioners should consider applying them in their own practice, where appropriate, to advance inclusivity and equity in technology design.

## 1 Introduction

Design is never neutral. As researchers, we did not have to look far to find evidence of queer marginalisation in youth digital mental health design. Here, *queer* is used as an inclusive umbrella term, consistent with research and inclusive language guidance concerning young people [1, 2], to describe a heterogeneous and intersectional group who are gender and/or sexually diverse. During co‐design research workshops with young people, conducted as part of our broader participatory research exploring youth digital mental health, our attention was repeatedly drawn to how digital technologies for mental health threaten to reinforce prejudice and exclude nuanced queer identities and experiences. Although the workshops focused on artificial intelligence in youth digital mental health and did not centre on queer identities, the young people reflected on digital technologies for mental health more broadly, asking why they often infer users' gender rather than enabling users to self‐identify their gender and pronouns. Further, they were concerned these technologies would likely demonstrate a binary view of gender and sexuality, perpetuating bisexual and transgender stereotypes and erasure. These reflections offered more than feedback, revealing the quiet, often unnoticed harm of digital exclusion of queer experiences. As digital technologies for mental health proliferate, who gets left behind by design?

## 2 How Technology Supports Youth Mental Health

We are in the middle of a global youth mental health crisis. Rising mental ill health is a multifaceted problem impacted by environmental, social, economic, political and technological change stressors and persistent stigma and discrimination about mental health [3, 4]. Young people (aged 12–25 years) are particularly at risk, as many mental disorders emerge during developmental stages [5] and are exacerbated by social challenges and inequities, including climate change, employment insecurity, wage theft and intergenerational inequity [3, 6]. The issue is further exacerbated by persistently overburdened services [7] and ongoing failures to implement evidence‐based support effectively and in a timely manner [8]. Among the many factors influencing youth mental health, queer experiences stand out as a well‐documented determinant, driven by persistent systemic failures at community, school and family levels to foster inclusivity, acceptance and belonging and to reduce institutional barriers and prejudicial sentiments experienced by queer young people across these contexts [9, 10]. Like other underserved groups, such as young Aboriginal and Torres Strait Islander Australians [11], young queer people encounter substantial mental health disparities [12]. Research shows that queer‐identifying youth in Australia experience high rates of depression, anxiety [13], suicidal behaviours and self‐harm [14]. The 2021 Writing Themselves In four surveys of 6418 queer young people in Australia (aged 14–21 years) found that 81% of participants reported high or very high levels of psychological distress, 78% reported having ever had suicidal ideation (58% in the past 12 months), 25% reported having ever had a suicide attempt (10% in the past 12 months) and 62% reported having ever self‐harmed (40% in the past 12 months) [15]. Furthermore, compared with heterosexual and cisgender young people, they face considerable difficulties accessing mental health professionals and peer support [16]. These trends are reflected on the global scale, with research indicating that queer young people are at elevated risk of poor mental health outcomes worldwide [17, 18].

Digital technologies for mental health promise improved outcomes for young people [19], as well as increased access, efficiency and scalability [8, 20, 21]. Today, young people use a range of digital technologies to access mental health resources, support and service pathways. These include internet search engines, online support services (e.g., BeyondBlue), phone services (e.g., Lifeline), social media platforms (e.g., TikTok and Instagram) and purpose‐built digital mental health technologies (DMHTs) or interventions. DMHTs include, for instance, mobile applications (apps) for mental health self‐management, interactive technologies (e.g., artificial intelligence chatbots) and immersive technologies (e.g., virtual reality) [11]. However, there is limited research at the intersection of diverse queer experiences and digital technologies for mental health [22], with only a small number of studies examining experiences involving, for example, queer‐positive school‐based mental health programmes [23], online knowledge‐seeking [24] and queer‐focused, online mental health services for young people living in rural/remote areas [16]. Worse still, non‐inclusive technology design practice persists, directly affecting the lives of queer young people.

## 3 Why Traditional Technology Design Practice Falls Short

Digital technologies, including those used for mental health support and access, often reflect non‐inclusive traditional design practice that overlooks the specific needs, values, expectations, patterns of engagement, user requirements and lived experiences of underserved communities. This problem stems from the persistence of conventional design practice (i.e., ‘the way design is actually done in real‐world design projects, rather than how design is prescribed’ [25]), which promotes greater utility rather than equity [20]. This is demonstrated by, for instance, online algorithms perpetuating prejudice against gender diversity [26], digital technologies continuing to emphasise gender binaries [27] and ‘biased by design’ big data and artificial intelligence solutions in health services failing to incorporate queer voices and account for health disparities, ultimately risking suboptimal outcomes [28]. Overall, these patterns reflect the pervasive embedding of heteronormative assumptions across health technology design [29].

Despite calls in the research literature and from national governments for greater personalisation and contextualisation of digital technologies for mental health [8], lingering, conventional technology design practice that configures the user as ‘everybody’ [30] excludes queer youth. This exclusion occurs through, for example, user research that treats identity as a single dimension rather than intersectional [31], homogeneous technology development team composition [32] and heteronormative assumptions in design decision‐making [33]. This manifests in, for instance, game‐based digital mental health interventions for young people being designed without features enabling users to customise gender and pronouns, thus failing to recognise gender and/or sexually diverse identities and expressions [34]. Although meaningful efforts to better engage queer communities in the design of digital technologies for mental health are reported in the literature, the extent of their engagement and the impact of their contributions on shaping these technologies are inconsistently reported [35]. Consequently, the potential for purposefully inclusive design in this space (e.g., Rainbow SPARX, which is a computerised cognitive behavioural therapy programme for queer youth [36]) to transform real‐world design practice at scale is limited. Ultimately, this widespread exclusion risks deepening existing inequities experienced by queer youth, disregarding their voices and arguably alienating them from digital mental health resources, support and service pathways. In addition, this exclusion threatens to miss opportunities to realise mental health benefits associated with enabling peer‐to‐peer support, diverse exploration of identity and expression, and safe, queer‐inclusive spaces via digital technologies [37]. Dealing with this issue at the root of technology design practice is critical to advancing digital mental health equity and accessibility for queer youth globally.

Although human–computer interaction (HCI) is essential to designing for successful user experiences, especially with DMHTs [38], research into the interplay of HCI, mental health and queer youth is limited. Moreover, the scarcity of queer representation in the design of digital technologies for youth mental health research reinforces existing social biases and stigma [39], thereby undermining effectiveness, relevancy, acceptability and uptake by design [30]. For example, design choices that embed gender binary prejudice in biased gender classification algorithms utilised on social media platforms, which this community often uses to access mental health information or peer support [40], may be inappropriately replicated across other digital technologies for mental health if design practice remains stagnant [26]. In fact, this issue is already evident in the design and evaluation of mental health apps, which persistently overlook gender and sexual diversity in both content development and visual representation [41]. The enduring self‐as‐user approach to HCI and technology design, which centres designers themselves as the key user, falls short of an equitable understanding of technology users [42]. This is particularly the case given the homogeneity of the information technology sector workforce, which remains dominated by ‘Global Northern, educated, middle‐class, white, heterosexual, young to middle‐aged men’ [31]. Without equitable technology design practice that appreciates diverse queer experiences, this community remains at the whim of apathetic and potentially harmful systems.

## 4 Breaking the Cycle

A substantial shift in design practice is needed to ensure greater inclusivity and equity when designing for queer young users. We propose three key principles for practitioners and researchers to consider when designing digital technologies used by queer youth to access mental health resources, support and service pathways: (i) partner and design with queer young people; (ii) embed queer theory into research and design; (iii) and design for sustainability.

### 4.1 Partner and Design With Queer Young People

First, it is essential to incorporate and survey diverse queer voices and experiences. At present, knowledge gaps exist at the intersection of queer youth, HCI and the design and use of digital technologies for mental health, with inadequate engagement with this community and an insufficient understanding of their relevant needs, values, expectations, attitudes and user requirements. Given that this area is underexplored and sensitive, the social and ethical considerations of these technologies within the queer youth mental health care ecosystem remain unclear and thus require investigation. For example, considerations include perceptions about potential added value and possible risks, as well as broader implications for care practice and policy. In line with best practice, researchers and practitioners should adopt a co‐design approach, utilising user‐centred and participatory design techniques to enact the principles of co‐production, within which co‐design represents one component [43]. Co‐designers should emphasise participation, collaboration and power‐sharing by directly involving queer young people in research and design processes and helping to create conditions for developing solutions that meet their needs [44]. Co‐design methods may include workshops, cultural probes, storyboarding, user personas, paper prototyping and think‐aloud activities. Online surveys and A/B testing can be used to support asynchronous, remote or anonymous participation while gathering sensitive feedback regarding identity and inclusivity and accessibility needs and preferences. Crucially, these methods also prioritise safety and privacy, which are key requirements for queer communities in digital mental health design [45]. In addition, these methods also allow for structured and quantifiable feedback on system requirements, such as design features, system functions, information flows, access modalities and user interface preferences, identified by queer young users. Recruiting young people to participate in these processes must account for intersectionality; that is, it must include intersecting identities, particularly those who are marginalised and thus often under‐represented, such as queer Aboriginal and Torres Strait Islander Australian youth, multicultural and multifaith queer young people or queer youth with disability. Importantly, meaningful inclusion of queer youth here requires adequate financial compensation for their expertise and time, as unpaid research and design participation and partnership risks perpetuating inequities [46]. Although this and the other principles proposed here are intended to be globally applicable, their implementation must be adapted to local legal, cultural and social contexts, particularly in settings where queer identities may be heavily stigmatised or criminalised and where safety, anonymity and data protection are paramount. As such, a broad, thoughtful, safe and purposive recruitment strategy is essential, seeking equitable representation of subgroups and intersecting identities, as well as incorporating recruitment through popular online social media platforms, trusted partners (e.g., inclusive health service providers, community organisations and social and advocacy groups), and recruitment services that support anonymous participation [44, 47].

### 4.2 Embed Queer Theory Into Research and Design

Second, we recommend embedding queer theory principles into research and design practice. Digital mental health needs *queering* to challenge the prevalence of heteronormativity and cisnormativity, confront rigid structures and norms underlying gender and sexuality in mental health and technology design, and better understand, empower and support queer young people's mental health [48]. In this context, queering can be achieved by:

- allowing research participants and technology users to assign their gender and pronouns with open‐ended responses to disrupt normative assumptions about identity;
- practising reflexivity and documenting any biases and gaps in representation identified to challenge biases in digital technology research, development and design; and
- leveraging alternate methods not typically applied to digital mental health, including story circles, body mapping and photovoice (documentation of experiences through photography), to incorporate non‐traditional epistemologies.

Crucially, queering requires appreciating the contextual and fluid nature of queer identity across diverse individuals, accommodating this heterogeneity where feasible, while recognising that designing for intersectionality is inherently iterative. When trade‐offs are necessary, design decisions and their rationales should be documented transparently, and mechanisms for iterative feedback and redesign should be developed. Furthermore, queering necessitates acknowledging that young people's technology practices continuously evolve, demanding critical examination of assumptions regarding usage patterns and accessibility. Embedding queer theory into the design of digital technologies for mental health in this way equips researchers and practitioners with the necessary knowledge, methods and tools to better accommodate and improve access for queer young people engaging with digital technologies for mental health [49].

### 4.3 Design for Sustainability

Third, designing for sustainability must be a priority to ensure the equitable and long‐lasting impact of digital technologies on health and well‐being. For often‐excluded communities, it is imperative to develop solutions that align with the sociotechnical systems in which they are deployed, consisting of existing and evolving perceptions, processes, policies, practices and sociocultural dimensions [50]. Designing for sustainability also requires authentic partnership and engagement with queer young users, communities and groups supporting queer young people, mental health service representatives, technology designers and developers, policymakers and standards organisations in design practice through co‐design methods, combined with a diversity, equity and inclusion lens, which in turn extends the reach of digital solutions to marginalised groups [50]. Sustainably designing digital technologies for mental health to benefit queer youth should prioritise context‐fit, co‐design, community partnership building, embedded lived experience researchers and meaningful alignment of ever‐evolving goals, priorities and values [50, 51].

## 5 Conclusion

Queer youth using digital technology for mental health remain at risk of invisibility, prejudice and exclusion perpetuated by conventional design practice. Therefore, queer‐inclusive, value‐sensitive technology design practice that seeks to minimise harm, maximise positive engagement and ensure safe, sustainable use of technology for mental health support, resources and service navigation is urgently needed to advance equity and accessibility. The imperative for inclusivity and equity in technology design does not stop at queer youth, nor in digital mental health. The three key principles outlined here must be foundational. By understanding and applying these principles through the lenses of queer youth and digital mental health, practitioners and researchers equip themselves with critical skills needed to embed equity, accessibility and justice into the broader design of digital technologies. Queering youth digital mental health marks only the beginning of a broader wave of transformative, inclusive innovation still to come.

## Author Contributions

**Adam Poulsen:** conceptualisation, methodology, investigation, writing (original draft), writing (review and editing), visualisation. **Ian B. Hickie:** writing (review and editing), supervision, funding acquisition. **Samuel J. Hockey:** writing (original draft), writing (review and editing). **Frank Iorfino:** conceptualisation, writing (original draft), writing (review and editing), supervision. **Haley M. LaMonica:** conceptualisation, writing (original draft), writing (review and editing), supervision.

## Funding

Ian B. Hickie is supported by a National Health and Medical Research Council (NHMRC) L3 Investigator Grant (GNT2016346). Frank Iorfino was supported by an NHMRC EL1 Investigator Grant (GNT2018157). Haley M. LaMonica was supported by funding from the Bill & Patricia Richie Foundation. This work was supported by a philanthropic funding donor affected by mental health who wishes to remain anonymous. The funders played no role in the planning, writing, design, data collection, analysis, interpretation, reporting or publication of this work.

## Disclosure

Not commissioned; externally peer reviewed.

## Conflicts of Interest

Ian B. Hickie is a Professor of Psychiatry and the Co‐Director of Health and Policy, Brain and Mind Centre, University of Sydney. He has led major public health and health service development in Australia, particularly focusing on early intervention for young people with depression, suicidal thoughts and behaviours and complex mood disorders. He is active in the development through co‐design, implementation and continuous evaluation of new health information and personal monitoring technologies to drive highly personalised and measurement‐based care. He holds a 3.2% equity share in Innowell Pty Ltd., which is focused on digital transformation of mental health services. Adam Poulsen, Samuel J. Hockey, Frank Iorfino and Haley M. LaMonica have nothing to disclose.

## Data Availability

This article includes no original data.
